# Comparison of human cell signaling pathway databases—evolution, drawbacks and challenges

**DOI:** 10.1093/database/bau126

**Published:** 2015-01-28

**Authors:** Saikat Chowdhury, Ram Rup Sarkar

**Affiliations:** ^1^Chemical Engineering and Process Development Division, CSIR-National Chemical Laboratory, Dr. Homi Bhaba Road, Pune, Maharashtra 411008, India and ^2^Academy of Scientific & Innovative Research (AcSIR), New Delhi 110 001, India

## Abstract

Elucidating the complexities of cell signaling pathways is of immense importance to gain understanding about various biological phenomenon, such as dynamics of gene/protein expression regulation, cell fate determination, embryogenesis and disease progression. The successful completion of human genome project has also helped experimental and theoretical biologists to analyze various important pathways. To advance this study, during the past two decades, systematic collections of pathway data from experimental studies have been compiled and distributed freely by several databases, which also integrate various computational tools for further analysis. Despite significant advancements, there exist several drawbacks and challenges, such as pathway data heterogeneity, annotation, regular update and automated image reconstructions, which motivated us to perform a thorough review on popular and actively functioning 24 cell signaling databases. Based on two major characteristics, pathway information and technical details, freely accessible data from commercial and academic databases are examined to understand their evolution and enrichment. This review not only helps to identify some novel and useful features, which are not yet included in any of the databases but also highlights their current limitations and subsequently propose the reasonable solutions for future database development, which could be useful to the whole scientific community.

## Introduction

Biochemical pathways, the molecular mechanism through which the cellular components are governed in the extra and intra cellular reaction networks, are involved in various physiological and cellular developmental processes and sensitive to the external or internal fluctuations of the cells (1). These pathways can be categorized into three major groups: metabolic, signaling and gene regulatory networks, which also coherently control the expressions of some set of genes, proteins or chemical compounds to regulate different phenotypic expressions to display distinct biological traits (2). Study of various biochemical pathways is therefore very much important to dissect their roles in several human diseases, such as cancer, diabetes and cardiovascular disease and in severe birth defects, such as placental and neurological defects (3–8). Hence, a comprehensive pathway map with detailed descriptions of different types of chemical modifications in the reaction cascades is required. Also, it is useful to discover the possible reaction paths, which are critical for the transitions of cellular states from normal to disease scenario.

However, initially the construction and analysis of such large and complex reaction networks were not so easy, as the unavailability of advanced tools and techniques for the functional annotations of various unknown or newly identified genes/proteins in the pathway diagram were the major problems to the pathway curators. Fortunately, the advancements in cellular and molecular biology experiments, high-throughput genomics or proteomics studies and the successful completion of human genome project flourished this field by generating plethora of genomics and proteomics data (9–11). Moreover, the sub-cellular localization data of the pathway components made it possible to annotate the pathway elements according to their locations in the constructed pathway diagram (12). Eventually, many communities, research groups and database developers got involved into the pathway reconstruction by collating experimental observations from published literatures and thus numerous different types of biochemical pathways were successfully mapped (1, 12). Various databases with interactive user-friendly interfaces are now developed to facilitate several operations, such as pathway data retrieval, sharing and storing process (13). This in turn helps to store these enormous amounts of constructed pathway data in a proper format and to retrieve easily across the internet.

Moreover, the importance of such databases is not restricted to the bench biologists for only accumulating the experimental data but also becomes valuable to the *in* *silico* model developers for mathematically interpreting the emerging properties of different cellular networks upon its exposure in various external cues (14). Dissection of the underlying complexities of different cellular and physiological functions by analyzing the activity profiles of various signaling pathways are receiving more attention to the researchers, and in this context, various databases are immensely contributing by providing the compiled experimental information of the required pathway (15). These databases are also serving the purpose of the identification of novel drug or drug targets for various diseases. Spatial annotations or compartmentalized pathway information of the bimolecular entities provided by these databases are also very much useful for the molecular and cell biology experiments to track the expression dynamics of various marker proteins *in* *vitro*. Besides, various mathematical models of signaling pathways also get huge inputs from these resources. However, the successful simulation of such *in* *silico* models are also dependent on the proper data inputs and can be achieved by using proper ontology while curating the data from various databases (15, 16). Depending on the architecture and data storage systems used by different databases, the procedures of pathway data visualization, access, storage and the analysis also differ significantly (17). As a result, it takes too much time to the users to manually extract the pathway data from these databases. All these impose a huge obstacle to develop a general and automated pathway data curation application, which may readily extract and compile pathway data from these databases. To overcome this problem, almost all the academic databases (e.g. REACTOME, PANTHER, NetPath, NCI-PID and SignaLink) and various pathway curation communities have developed computer readable, easily accessible, standard file formats, such as Systems Biology Markup Language (SBML), Biological Pathway Exchange (BioPAX), System Biology Graphical Notations (SBGN) and PSI-MI, to accelerate the data sharing process across various platforms (18–21). However, the information related to such facilities, challenges or limitations in different databases is still not available yet and, hence, demands a proper documentation on this broad topic.

To guide the database users, a good review with the detailed descriptions and scopes of the existing pathway databases is absolutely required. However, several reviews on the biochemical pathway databases are published in the literatures focusing on the analysis of cell signaling pathways and are found to be more vocal toward the discussions of the challenges that have been faced by the authors while collating and normalizing the pathway data from various resources. Meanwhile, few review articles, dedicatedly focusing on the metabolic pathway databases, are also published by highlighting their pros and cons, whereas a very few discuss on both metabolic and signaling pathway databases (22–27). Besides these reviews, the benefits, limitations and the challenges of few signaling pathway databases, mainly KEGG (1), REACTOME (28), PANTHER (29), NCI-PID (30), WikiPathways (31) and associated tools in the context of data collation for developing dynamic or quantitative model of signaling pathways are also reviewed (14). Moreover, the limitations related to data searching and easiness of using the six major databases: REACTOME, Pathway Commons (32), KEGG, InnateDB (33), NCI-PID and WikiPathways for *in* *silico* model development are also nicely compared in the previously published review (17). But a comparison of the well-known and broadly used cell-specific signaling pathway databases with detailed discussions related to their current limitations, data types, data access policies and the available technical features is still not available. Hence, this review article is intended to recapitulate those aspects by considering not only the major signal transduction databases but also the available sources through which one can access the pathway data.

## Scope of this review

According to the pathway resources repository, Pathguide (13) and *Nucleic Acid Research* Database Issue, the rate of publication of human cell signaling databases each year is not negligible, rather it is quite impressive. However, because of some unknown reasons, few published databases could not keep the pace with the current requirements and eventually found inactive while accessing their HTTP links. Hence, before starting this review, it was necessary to short list the databases, which are active till this date. To select the database and to restrict the scope of this review, two criteria were set up for the comparison: (i) the database should solely or partially provide the human cell signaling-related data and (ii) the HTTP links of the databases should be in active or working state and can be readily accessible to the users without any accessing charge. At the time of communicating this review article, there were altogether 24 such active, open source, well known and broadly used human cell signaling databases available, which fulfill these two criteria. In the Supplementary Table S1, the name and the HTTP links of these databases are provided for the interested readers. It should be noted that there are few databases included in this review article for comparison, such as Pathway Commons and GOLD.db, which do not satisfy the quality of a primary database as these databases do not posses any self-curated pathway data (32, 34). However, the data possessed by these databases are the processed data of the other primary databases, which in turn facilitates the experimental, computational biologists and the software developers to extract, collate, analyze and modify the pathway data. Moreover, there are few databases, such as SPAD, GOLD.db and DOQCS, included in the comparison list, which were updated long back, but their HTTP links are still working and can be easily accessible to all the database end-users. Hence, it is also necessary to consider these databases. Similarly, BioModels and DOQCS are also considered in this review though they are not same as the other signal transduction databases. These two databases are thought to be the pioneers as they provide the quantitative model to build the dynamic models of various important signal transduction networks and thus can help to shed the light on the progress of mathematical modeling of signaling pathways. Hence, to draw the attention of a wide spectrum of research areas and the database users, this review has selected these 24 well-known and broad human cell signaling-related databases for further comparison. As aforementioned, this review not only intends to compare the major databases but also searches and compares all the possible and related resources of signaling networks, through which one can get human cell-specific pathway information for the purpose of various signaling pathway-related research works. However, it is also worth to mention that to restrict this review in a particular field of interest and due to the space constrain, different other similar types of databases [such as Signal Transduction Knowledge Environment (STKE), HPRD and BIOGRID], which provide the connection maps, protein–protein interaction (PPI) and small molecule interactions data of signaling pathways, are not considered in this review (35–37). Although in certain cases, to highlight some useful features of these databases, this review has cited the examples from these resources.

In short, the scope of this review article is mainly focused on the review and comparisons of these 24 databases including the six major databases mentioned earlier. Moreover, a brief history and evolution of these selected databases are discussed, and subsequently, several aspects of these databases by comparing their pathway information and the in-built technical features are also reviewed. This will help to analyze the current situations of the databases with respect to the present requirements in biological research and at the end will help to distinguish the merits and demerits of the database with respect to one another and to identify some novel and useful features, which are not yet included in any of the databases till now. Moreover, this comparison will also help the database end-users (both experimental and theoretical biologists) to acquire the knowledge of various resources from which they can collate different types of signal transduction information. To elaborately discuss this section, detailed comparisons of different types of available pathway data, which are present in different databases, are discussed for the interest of database end-users. It should be noted that the comparison discussed in this review are completely based on the open access data, which are provided by all the databases included in this review. The quality of data can be varied between open access and the purchased or licensed versions, but comparing those aspects is beyond the scope of this review. Moreover, to compare the data heterogeneity across different databases, a case study on human Hedgehog and Notch signaling pathways are also discussed. Simultaneously, the facilities, their limitations and challenges regarding various technical features involved in these databases are highlighted and the possible way out from those constrains are also proposed for future database development. Hence, the current review is intended to help both the database end-users and the databases developers by providing the comprehensive comparisons of the data contents and the technical features available in the selected databases.

## History and evolution of the databases

The history of biochemical pathway databases starts from the construction and visualization of metabolic pathway maps by the researchers of various laboratories. Initially, these metabolic pathway maps are used to be referred as ‘Biochemical Pathways Wall Chart’, originally developed by Dr. Gerhard Michal, on which it was difficult to locate any particular enzyme or metabolite (23). In the last two decades, the advancement of computer science, internet, web browsing and data sharing policy has made it possible to host and share the rendered pathway images through the web browsers. In 1993–94, EcoCyc, a family of Cyc family database launched the first formal representation of metabolic pathways of *Escherichia* *coli* (38, 39). Subsequently, in the year of 1995-96, the database ‘Kyoto Encyclopedia of Genes and Genomes (KEGG)’ was launched by Prof. Minoru Kanehisa, Kyoto University, Japan, which initiated the web hosting of manually curated pathway diagrams of metabolic, genetic and signal transductions networks of different organisms. It was the first initiative, where the binary gene interaction data from genome projects was used to map and group with functional dependencies and was subsequently presented by so called higher level information schema or pathway diagrams (1).

**Figure 1.**
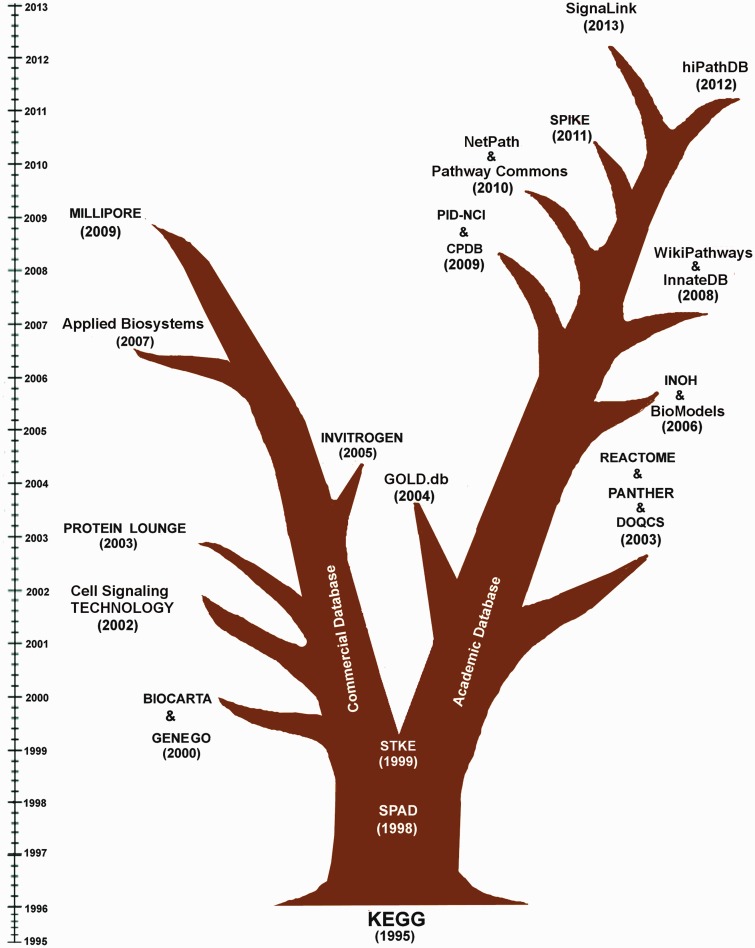
Evolutionary tree of the existing and active signaling pathway databases. This figure clearly depicts that KEGG, SPAD and STKE can be thought as the pioneers in the field of the development of human cell signaling databases. In the subsequent years, the evolution of human cell signaling databases is mainly led by the ‘Commercial Groups’ and several ‘Academic Groups’. The first commercial database, BIOCARTA and GENEGO were launched in 2000. In the subsequent years, several databases, like Cell Signaling TECHNOLOGY (2002), PROTEIN LOUNGE (2003), INVITROGEN (2005), Applied Biosystems (2007) and MILIPORE (2009) were also started to provide human cell signaling data freely to the users. By integrating the signaling pathway components with corresponding antibodies, drugs and inhibitor molecules, these commercial databases are promoting the bench biologists to order and purchase those products more conveniently from their websites. On the other hand, the evolution of the other branch of the human cell signaling database is been continuously developed and monitored by several academic research groups across the world. Since 1995, almost each year, on an average one or two such databases are launched. The objectives of these databases are wider than the commercial databases and are not only restricted to the pathway data annotation and presentation but also to the analysis of cross talks of multiple pathways, drug target identification, *in silico* simulation, development of computer readable pathway data sharing process and pathway analysis, etc.

Inspired by this approach, several other academic and commercial groups put their efforts to construct various biochemical pathways from the experimental data (13). As time proceeds, a group of databases restricted themselves only to curate either metabolic (e.g. BioCyc and MetaCyc) or gene regulatory (e.g. TRED) or cell signaling networks (e.g. SPAD and NetPath) (40–44). In this article, the history and evolution of human cell signaling databases, which are found active till this date, are only discussed. During last two decades, the evolutionary progress of these databases is clearly divided into two branches, lead by Commercial and Academic research groups, and are shown in Figure 1 (generated using the respective publication or launching date whichever is available in the literatures or in the web site). It is worthy to mention that before 1998–99 (after the publication of KEGG), there were no such divisions or progress observed to develop human cell signaling databases for commercial purposes by any commercial groups or companies. Hence, KEGG, SPAD and STKE/Science Signaling Database (which are made for academic purposes) are placed in the middle of the database evolution tree in Figure 1, and after STKE (1999), the history of human cell signaling databases is bifurcated into two branches: Commercial and Academic database. Following 1999, the commercial databases GENEGO/METACORE and BIOCARTA (2000) started to provide signal transduction data to the common users. However, BIOCARTA started the pathway curation on the basis of ‘open source’ approach, GENEGO was more inclined to merchandise their pathway data by providing a large collection of pathway knowledgebase and various commercialized pathway analysis tools. However, a portion of its data contents (i.e. sample pathways) are also available freely in their web sites for better users' experience and publicity purpose. In this respect, it is also important to highlight another pathway analysis tool, Ingenuity Pathway Analysis (IPA), which has also the same functionalities and a comprehensive knowledgebase of biological networks with respect to the GENEGO/METACORE pathway analysis and database service. There are many analysis tools currently present in IPA to perform various types of network analysis, gene expression studies, toxicity checking and microRNA target filtering. However, it does not provide any sample or free pathways in their web site, and hence, this commercial knowledgebase is not included in this review for the analysis of its quality of pathway data contents. From Figure 1, it is also depicted that the commercial branch of the database evolution tree is then successfully led by other various commercial databases, such as PROTEIN LOUNGE and Cell Signaling TECHNOLOGY.

Although, as expected due to the commercial policies of these databases, it is observed that during this time period, the objectives of the commercial databases are confined only to the pathway image rendering, its commercialization (except BIOCARTA) and their analysis through various commercial software, whereas the academic databases are more inclined to the inclusion of open source pathway analysis tools, sharing of computer readable pathway data for *in* *silico* study, flexible file formats for inter database data sharing purpose, development of uniform pathway annotations and formats, etc. After KEGG, the major impact on this field of databases development was made by STKE, developed by American Association for the Advancement of Science and Stanford University Libraries (35). Although it represents the pathway maps in a different way (i.e. protein–protein connection or interaction maps) compared with the other similar databases, the importance of this database in the history of the progress of cell signaling database development is not negligible. It was the first initiative, which started to provide the tools for compiling and organizing the pathway information in the cross-disciplinary field of signal transduction. It showed the way to populate the database contents by the experts of pathway editors or ‘pathway authorities’ and to share the data to the registered users at free of cost (35). To perform that, the technical team of this database created a common platform called CMADES, by which the pathway editors could manually enter the connection map of a signaling pathway and automate various tasks such as keeping the record of data entry and pathway reconstruction. Eventually, the academic databases, such as KEGG, PANTHER, REACTOME and Pathway Commons (32), are also started to provide similar types of tools to the pathway curators and database developers, and in this context, various API services and the computer readable pathway data sharing files, like SBML, BioPAX, KGML, etc., are distributed for academic purposes to the non-commercial users and software developers at free of cost (18, 45–47). For the sake of readers' interest, it is noteworthy to mention that recently KEGG has started charging and asking licensing fee to all the commercial and non-commercial users through separate licensing agreement to download its data contents through FTP (File Transfer Protocol). However, for visualizing the pathway maps, genomics, reactions or other types of data though their HTTP link is completely free and does not require any license or subscription fee. KEGG API can also be used for searching and computing the data from the database, except for the bulk download. Individual download of KGML (a specific XML-based data sharing file format made by KEGG developers) file for a particular pathway can also be possible through its HTTP link. Hence, it is worth to mention that most of the pathway databases are being evolved in such a way, so that it can provide maximum information and facilities to the users by smoothly sharing the data contents across a large number of users. Moreover, there are many other features, which these databases have included to facilitate the users' experience to interact the database throughout the progress of evolution. However, there are few constrains, which remain unsolved and still demand the attentions of the developers and the end-users of this databases. To make these databases more resourceful and user friendly, and to highlight the constraints and limitations, a comprehensive review on the basis of comparing individually their available features is utmost required.

However, one can also think to compare the databases by grouping them in different categories on the basis of other criteria. For example, the signaling databases can be classified according to their mode of collating pathway data and can be grouped as ‘Primary or Self-curated’ database, ‘Secondary or Aggregator’ database and 'Hybrid' database, which possess both the self-curated and aggregated pathway data. Major academic databases KEGG, SPAD, DOQCS, NetPath, REACTOME, SignaLink, SPIKE, BioModels, INOH and PANTHER, and almost all the commercial databases BIOCARTA, GENE GO/METACORE, Cell Signaling TECHNOLOGY, PROTEIN LOUNGE, MILLIPORE, Applied Biosystems and INVITROGEN fall into the first category. All these databases contain manually curated data, which are mostly curated by the experts of this field. On the other hand, the academic databases Pathway Commons and hiPathDB can be classified in the second group. Pathway Commons integrate the pathway data from the signaling databases, REACTOME and NCI-PID. It also curates PPI data from BioGRID, MINT and HPRD, etc. Moreover, hiPathDB collates pathway data from KEGG, BIOCARTA, NCI-PID and REACTOME databases and subsequently removes the redundancy to provide a unified and reformatted pathway models. Other useful databases, such as WikiPathways, NCI-PID, GOLD.db, CPDB and InnateDB are dependent on self-curated as well as aggregated data from the primary databases and hence fall into the 'Hybrid’ database category. WikiPathways contains both manually curated data (provided by the pathway curators) and imported pathway data from NetPath, KEGG, etc. In NCI-PID, 137 human pathways (or 9248 Interactions) are manually curated by NCI-PID administrators (up to September 2012), whereas 322 human pathways (or 7575 Interactions) are directly imported from BIOCARTA and REACTOME databases. There are only three self-annotated pathways found in GOLD.db, whereas the information of other pathways is mostly collated from KEGG and BIOCARTA by the GOLD.db database developers. Similarly, InnateDB contains its own self-annotated pathway data and the imported pathway information from other resources, such as KEGG, NetPath, INOH, REACTOME and NCI-PID. It is also worth to mention that several other primary databases, such as Uniprot, Genbank, GO database, gene expression databases (GEO, EBI-Array Express) also annotate the pathway-specific information in their annotated data sets, which sometimes prove to be beneficial for the researchers in this field.

## Database comparison

The purpose of the comparison of all the selected 24 databases, on the basis of individual features, is to review the current scenarios of the present cell signaling databases by considering the current requirements of the pathway analysis as reference point. It is previously mentioned that this comparison is not intended to compare the databases at particular direction or to a specific community of signaling databases, rather it addresses the available facilities, challenges, limitations and their reasonable solutions for these databases, which are faced constantly by both the database end-users and the database developers nowadays. However, to organize this broad topic, the entire comparison is divided into two parts: ‘Data contents’ and ‘Technical features’. It is a well known fact that the developers of any database mainly focus on two different aspects, firstly, the 'primary or raw data’ and/or the ‘secondary or processed data’ and secondly, the ‘technical operations’ through which a database can be smoothly accessed, queried and several applications can be run. Hence, to compare the databases, it is chosen to focus on these two major characteristics: (i) Pathway Information or data and (ii) Technical Details. In the subsequent sections, all the 24 databases under study are compared according to the various features within these two major categories. Moreover, to see the pros and cons of these features in real time scenario, simultaneously a case study is also performed on Hedgehog (6, 48) and Notch signaling pathways (49, 50).

### Comparison based on pathway information

#### Comparison of different signaling pathway data.

**Figure 2.**
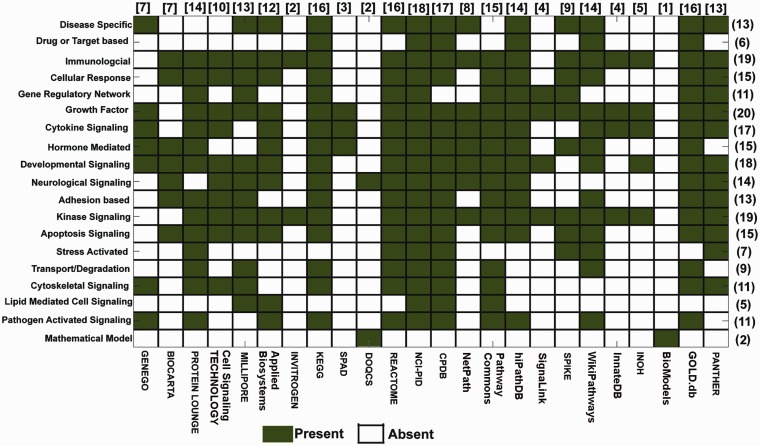
Signaling pathway databases and the available pathway data types. This figure illustrates a matrix, whose ‘Rows’ and ‘Columns’ represent the number of different types of signaling pathways (*Y* axis) available in different databases (*X* axis), respectively. Color legends are used to represent the ‘presence’ or ‘absence’ of different types of signaling pathway data (total 19) available in the selected 24 databases. The numbers in first bracket indicate the number of the databases, which contain a particular type of pathway data (represented in row wise), whereas the numbers in square bracket indicate the total number of different types of pathway data present in a particular database (represented in column wise).

Various types of cell signaling pathways, such as developmental (e.g. Hedgehog and Notch), disease specific (cancer, cardiovascular, etc.), immunological (T cell, B-cell signaling, etc.) and apoptotic, function in human body throughout the embryonic to adult stages. In total, 19 such broad classifications of pathway data types are found across all the databases. In Figure 2, a matrix is presented to show these classifications against the databases to show how many different types of signaling pathways are presently available in various databases or *vice* *versa*. The different data types and the number of pathways calculated to construct this matrix representation are taken from the open access data presented in all the databases (i.e. both the commercial and academic databases). However, it should be taken into the consideration that the pathway data provided by these commercial databases may vary in both quantitatively and qualitatively between the open access and purchased modes. For example, the pathway information provided in the database PROTEIN LOUNGE is the reduced version of the main pathway information, which they provide only to its subscribers. According to this figure, NCI-PID has the highest number (total 18) of different types of signaling pathways present in its database, whereas BioModels (51) and DOQCS (52) contain lowest number (only 2) of different types of pathway data. Moreover, including its own curated data, NCI-PID also hosts the pathway data, which are gathered from other two major sources: BIOCARTA and REACTOME. On the other hand, BioModels (dedicated for quantitative models of biological processes) and DOQCS (a resource for neurological signaling pathways) have a very specific type of dedicated pathway data present in the database, whereas the major databases such as CPDB (53), KEGG, REACTOME, GOLD.db (34), PROTEIN LOUNGE, MILLIPORE and PANTHER (29) are the resources of signaling pathway data, which contain a wide spectrum of pathway data in their repositories. This comparison clearly indicates that NCI-PID, KEGG, CPDB, etc. are the largest hub or source for different types of signaling pathway data, whereas BioModels, DOQCS, SPAD, etc. are very specific for a particular type of pathway data (e.g. mathematical rules of the pathway reactions, kinetic parameters and concentration values). Hence, it is worth to mention that the different types of signal transduction pathways and their corresponding annotations presented in these databases are comparably lower than the other general databases. Although to give the information about the resources of the quantitative data of signaling pathways to the interested users, it is also relevant to discuss these databases in this context. This comparison also shows that depending on the requirements, a user can select any of the databases for manual pathway curation, and the application developers can also use this information to create software for efficient and fast data curation from different sources.

Figure 2 also depicts the type of signaling pathways, which are commonly or rarely found in different databases. For example, in Figure 2, it is shown that ‘growth factors (e.g. EGF, IGF etc.)’ -induced signaling pathways are commonly found in almost all the major and widely used databases. Including this, other types of signaling pathways, such as immunological, developmental and kinase signaling pathways, are also commonly found in most of the databases, whereas the drug- or target-based, stress-activated or lipid-mediated signaling pathways are rarely found in few databases, such as REACTOME, CPDB, NCI-PID, WikiPathways and GOLD.db. This finding clearly indicates that the curations of these types of signaling pathways are overlooked by most of the databases till now. It is also worth to mention that the mathematical model of the signaling pathways is found only in BioModels and DOQCS, which have large numbers of model files and kinetic data stored in its repository and can be used to simulate various signaling cascades *in* *silico*. BioModels provides the model files in various computer readable file formats such as SBML, Octave (m-file), Scilab and BioPAX, whereas DOQCS provides the model files in GENESIS file format (51, 52).

#### Pathway annotation and nomenclature.

To provide easier access and better query procedures to the users, a database should index its stored data in more organized fashion. However, proper indexing of such large data can be achieved, if the pathway annotation and nomenclature are properly followed. It is observed that although the databases assign some entry number to each pathway, there is no such specific nomenclature exists for assigning the name of the signaling pathways in the databases. The ambiguity of the pathway nomenclature may sometime confuse the users to select the appropriate pathway within the databases. For example, in case of Hedgehog and Notch pathways, KEGG, PANTHER, WikiPathways, etc. are using common names such as ‘Hedgehog signaling pathway’ and ‘Notch Signaling pathway’, respectively, but others use different nomenclatures (e.g. BIOCARTA assigns the Hedgehog pathway as ‘Sonic Hedgehog (Shh) Pathway’ and NCI-PID as ‘Signaling events mediated by the Hedgehog family’). One of the reasons behind the heterogeneity of the pathway nomenclature is that there is no standard naming convention exists for the biochemical pathways. Moreover, assigning an appropriate name for a particular pathway is another problem for the database developers; as an example, in case of Hedgehog pathway one can assign its general name ‘Hedgehog Pathway’, but the other can assign its name on the basis of the main proteins of this pathway, such as ‘GLI mediated Hedgehog signaling pathway (54)’ or ‘SHH mediated Hedgehog pathway (55)’. Here, SHH and GLI are the ligands and target transcription factor of Hedgehog pathway, respectively. Similar examples are also found for other pathways where the name of a pathway is given either on its ligand, receptor or main target transcription factor. Because of this reason, different names of a same pathway can be found in different databases, and sometimes such ambiguity can cause a serious challenge to the researchers and to the pathway-based application developers for further searching and curating pathway data from internet. Hence, a standard nomenclature of the pathway entries is required.

**Figure 3.**
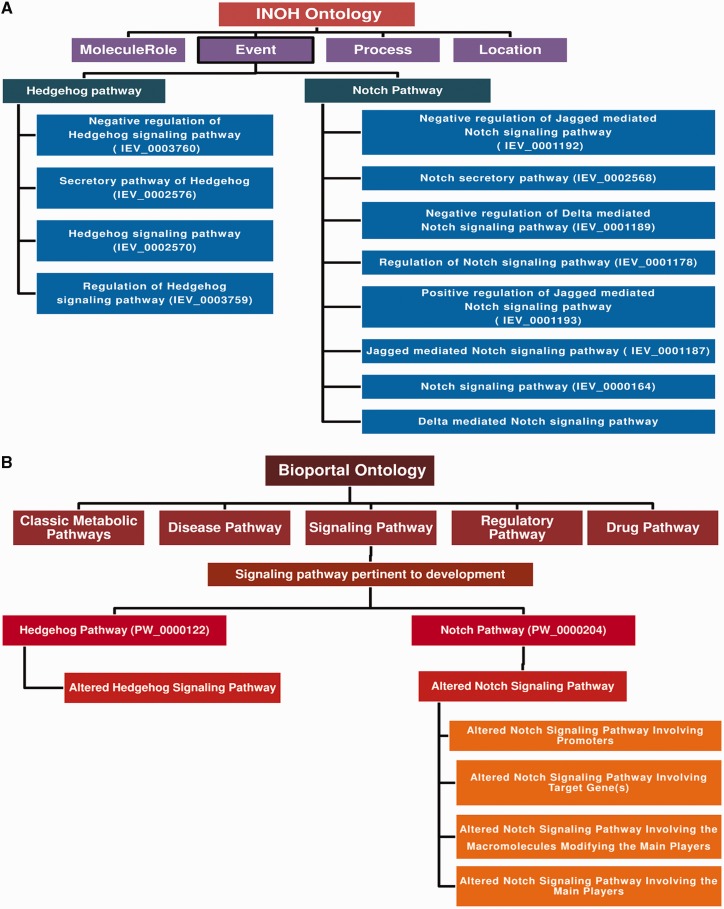
Pathway ontology used by INOH and Pathway Ontology portal. (**A**) INOH-based ontology uses the ontology or hierarchical formats to annotate the curated signal transductions and metabolic pathways in BioPAX and INOH formats. ‘MoleculeRole’, ‘Process’, ‘Location’ and ‘Event’ are four types of ontological terminologies, which are used by this database. Under the ‘Event’ category, all the pathways are annotated and each pathway is then further categorized into several sub-categories according to their molecular functions. For example, the Hedgehog pathway is divided into four sub-categories: ‘Negative regulation of Hedgehog pathway’, ‘Secretory Hedgehog pathway’, ‘general Hedgehog pathway’ and ‘Regulation of Hedgehog pathway’. For each category, there is also a unique ID number present, which classifies a pathway according to its various molecular functions. (**B**) ‘Bioportal: Pathway Ontology’ (http://bioportal.bioontology.org/ontologies/PW) forms the ontological tree by using the conceptual terminologies: ‘classic metabolic pathway’, ‘disease pathway’, ‘drug pathway’, ‘regulatory pathway’ and ‘Signaling pathway’. The signaling pathway is further divided into various categories, such as ‘calcium mediated’, ‘adhesion based’, ‘G-protein mediated’ and ‘Developmental process’. Hedgehog and Notch pathways are kept under the ‘signaling pathways pertinent to development’ category. Furthermore, a unique ID is provided for each pathway with its sub-categories in the ontology tree. Here, the altered pathways for each pathway is also considered and kept under different classification, which in turn helps to distinguish the normal and its altered counterpart. For example, in case of Notch signaling pathway, it is classified as the possible alteration of Notch pathway by several factors such as by promoters, target genes and macromolecules and each altered pathway are assigned with different ID numbers.

‘Ontology (i.e. structured vocabulary or the terms for conceptualization)’-based pathway annotation and nomenclature can be a reasonable solution for this problem. By forming the ontology-based pathway annotation tree, one can easily assign a specific name and the functional annotations for a particular pathway in the database. There are two such types of ontology database exist: INOH (56) and BIOPORTAL: PATHWAY ONTOLOGY. In Figure 3, the detailed descriptions of these two ontologies are briefly discussed. The database developers can follow any of this ontology to annotate and give appropriate name to the pathway of interest. Once a pathway is annotated in the ontology tree, then the pathway can be assigned by a particular ontology number, which will be its universal accession number. On the other hand, the databases, such as KEGG, REACTOME and PANTHER, are using their own ontology to assign the pathways, but they are not normalized yet as the formation of such ontology evolved in a discrete fashion in different databases. Besides, these ontology formats are still not accepted by many database communities, and hence it requires more discussions and debates to introduce a full proof, authentic, well-established and widely accepted standard pathway ontology.

#### Data heterogeneity.

**Figure 4.**
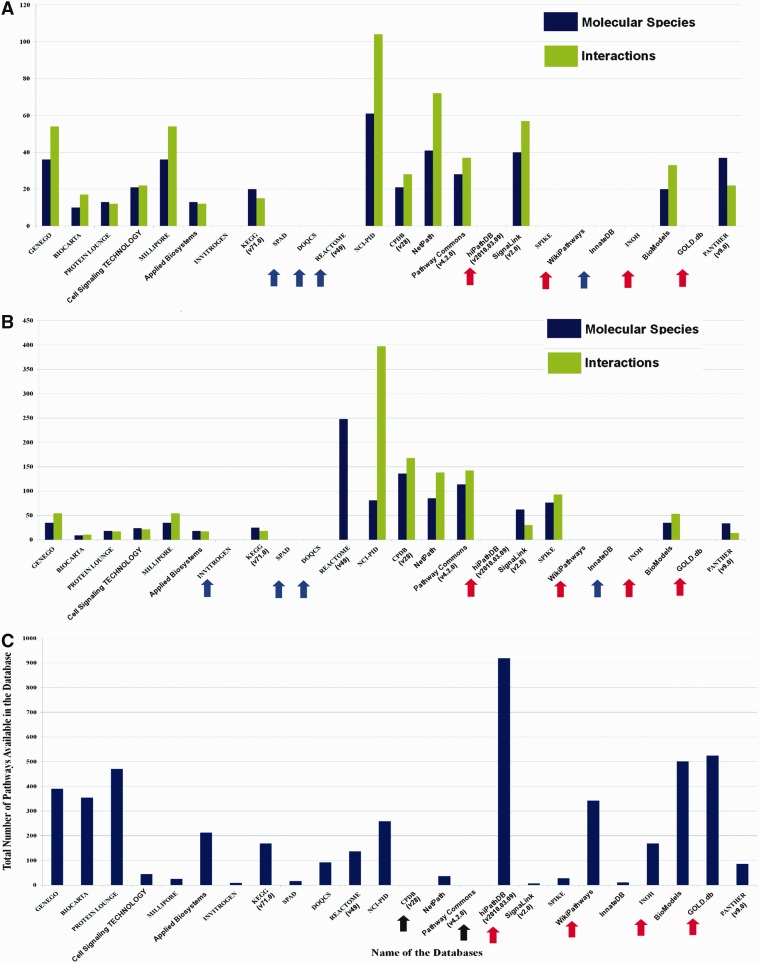
Pathway data heterogeneity across different databases. (**A**) and (**B**) show the comparison of the number of molecular species and interactions of Hedgehog and Notch signaling pathway across different databases, respectively. The names of the databases are presented along the *X*-axis with their corresponding version number (if available), mentioned in first parenthesis. Pathway statistics presented in these graphs (*Y*-axis) are taken from those corresponding database versions, i.e. on or before 21 July 2014. The blue arrows indicating the names of the pathway databases signify that these databases do not possess Hedgehog or Notch pathway information. Moreover, the databases indicated by red arrows show the databases that do not possess their own curated Hedgehog or Notch pathway data. These types of databases (e.g. WikiPathways, hiPathDB and GOLD.db) are mostly dependent on the curated data of other databases and therefore the data from these databases are not included in the comparisons. Although these databases possess the same data, which is available in other databases, but the importance of such databases are found in other aspects of pathway data analysis. (**C**) The total number of signaling pathways of each database is plotted and compared. Here, the black arrows indicate the names of the databases whose total number of available signaling pathway data could not be counted. It shows hiPathDB has highest number of available pathway data present in its database, though the data it contains are all gathered from KEGG, REACTOME and NCI-PID.

The variations in data curation by the developers make the distribution of pathway data in different databases more heterogeneous in nature. To examine the extent of this data heterogeneity, a case study on Hedgehog and Notch pathway is performed by manually counting the number of molecular species (i.e. proteins, genes, metabolites or the compounds/complexes) and interactions (i.e. biochemical reactions, chemical modifications, physical interactions, translocations and diffusion) available across different databases (Figure 4A and B).

This comparison shows that in case of both of these pathways, NCI-PID (an academic database) contains the highest number of molecular species and interactions in its database, whereas GENEGO and MILLIPORE provide more information when compared with the other commercial databases. Besides, the other databases, such as NetPath, CPDB, Pathway Commons, PANTHER and BioModels, also serve as the major resources for the curation of these two pathways (Figure 4A and B). These distributions of molecular species and reactions of Hedgehog and Notch pathway across different databases clearly show how the pathway information is heterogeneously scattered in different databases.

It is also observed that the heterogeneity of total number of signaling pathways (human) in different databases is seemed to be widely varied in different databases (Figure 4C). As this review article focus only on the human cell-specific signaling pathways, therefore this comparison will not account the metabolic or the biosynthesis pathways, which are currently present in the databases. Only the types of pathways, mentioned in Figure 2, are considered and counted for the total number of pathways across different databases. This comparison shows that BioModels contains the highest number of curated pathways followed by PROTEIN LOUNGE. In the category of commercial databases, GENEGO, BIOCARTA and Applied Biosystems have significant number of pathways, whereas KEGG, REACTOME, NCI-PID and WikiPathways contain higher number of pathways in the category of academic databases. However, it is worth to be noted that the molecular species and interactions available for Hedgehog and Notch pathways in BioModels or PROTEIN LOUNGE are comparably lesser than NCI-PID, NetPath and SignaLink (57). This characteristic is also true for the other pathways as well (comparison not shown).

To find out the reason of this data heterogeneity, it is required to know the sources from where most of the databases extract the data and the procedures through which these data are extracted from those sources. It is observed that despite the presence of several data sources, till now the most reliable and widely used resource is the experimental data available in the published literatures. Most of the primary databases, such as KEGG, REACTOME and PANTHER, are developed on the basis of manually curated pathway data from the experimental data published in literature or any other related resources. Hence, it is observed that to curate the pathway-related information, manual curation is the most accepted procedure till now. Simultaneously, it is also important to note that the accuracy and the reliability of this procedure depend on the intelligence of human resources, who are involved in the manual curation and extraction of pathway information from the large pool of experimental data. As a result, this manual curation procedure has both advantages and disadvantages. The advantage is that the prior knowledge of manual data curation, such as biological interpretation, reasoning and the syntax, which are highly required to curate the pathway information and reconstruction of new pathway map, can only be achieved through this process. On the other hand, the major drawback of this method is that the pathway data is not available from a single experimental resource as it is highly dispersed in multiple published articles, and hence, to extract the valuable information from this fragmented data source, a large number of human resources are required to be involved throughout the entire process. Significantly, it is obvious that the strength of human resources of different databases is not homogenous, and thus, the depth of the data curation varies significantly across different databases. Moreover, because human intervention is widely involved in this method, therefore the data contamination due to human errors can be another disadvantage and can lead to the serious error propagation to other resources and experimental findings.

However, because of its high level of authenticity and acceptability, till now manual curation is regarded as the best method for the pathway database curation when compared with the other methods like computational data curation, homology-based pathway reconstruction, genome mapping, comparative genome analysis, etc. (58–61). One of the major advantages of manual data curation is that it involves the scientists, experts and research scholars of this field for further review of the uploaded pathway data in the database. As a result, it always gets the chance for additional refinements, if the rigorous monitoring and smooth communication between the curators and the reviewers are provided properly. Updating and sharing of newly identified species and/or interaction across the major databases and its thorough revision by the reviewers can also be used to increase its authenticity across the pathway communities. Another, possible way through which the authenticity of the manually curated data could be verified further is by the aggregators and/or various hybrid databases. These databases (e.g. Pathway Commons, GOLD.db, InnateDB, CPDB and hiPathDB) aggregate the data from various primary sources (e.g. KEGG and REACTOME) and process the data for further pathway analysis. As these databases are collating the data from various resources, hence these databases have the chance to share more number of pathway data to a larger community of pathway database end-users as well as can reduce the chance of error propagation of the primary database by further cross checking the primary raw pathway data. Moreover, as these databases are integrating and processing the data of various major databases, hence there is an option to reduce the redundancy and heterogeneity of the collated data by further filtering and normalizing the aggregated data to make homogenous, information-rich and comprehensive pathway maps of signal transduction network. In this context, one can also consider the example of manual curation and data sharing process used by BIGG database, which integrate the huge collection of several published genome-scale metabolic data into a single location and further uses standard nomenclature to share those data across a large number of database end-users communities. This review also demands the development of similar web-based resources of signal transduction data. CPDB has already started this process and is providing seamless interaction network of signaling pathway. Currently, it includes 32 different types of resources for data aggregation including their own curated data and reconstructing various types of signal transduction network.

#### Cross references of the signaling reactions.

**Figure 5.**
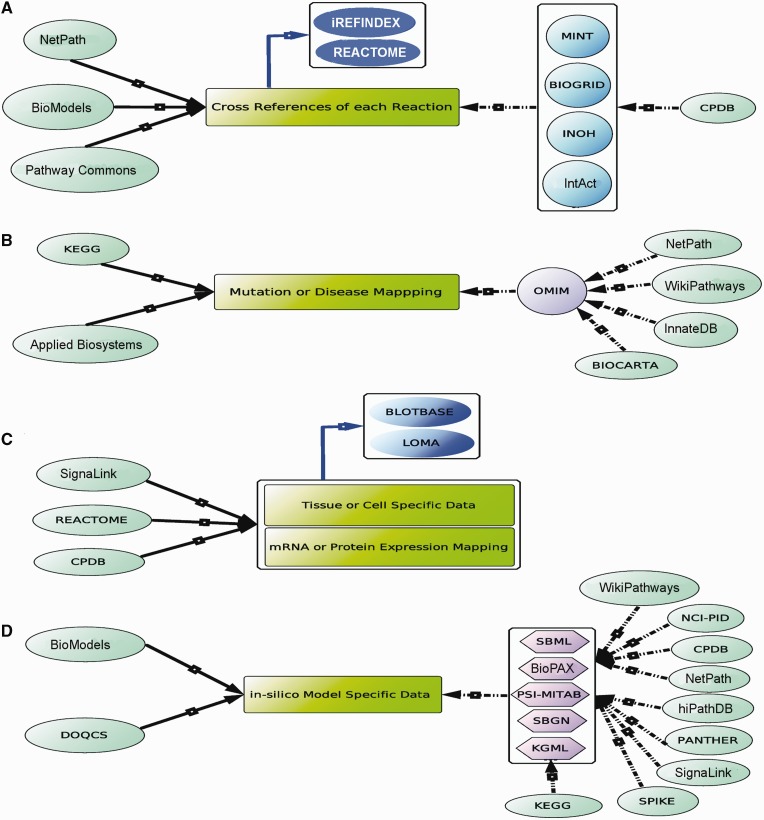
Existing and proposed cross referencing of pathway information. The arrows, black, dotted black and blue, signify the manual curation, hyper-links and proposed cross-referencing (hyper-linking), respectively. (**A**) NetPath, BioModels and Pathway Commons manually annotate each interaction of a pathway, whereas CPDB uses other PPI databases (e.g. MINT and BIOGRID) for the annotation of each reaction. On the other hand, iRefIndex, a non-redundant protein interaction database can also be used to annotate the interaction with its specific ID. (**B**) KEGG and Applied Biosystems have their own collated data to map the disease-specific information with the pathways, but the others, like NetPath, WikiPathways, BIOCARTA, etc., use another disease database OMIM. (**C**) SignaLink, REACTOME and CPDB provide the manually collated tissue-specific m-RNA or protein expression data along with the pathway information. Here, it is also proposed that the data from the databases, like BLOTBASE and LOMA, can also be used to annotate such type of data with the pathway information. (**D**) The manually collated *in silico* model including rate parameters and kinetic data is provided by only two databases, BioModels and DOQCS, whereas to provide only the reaction graphs or schema, computer readable files, such as SBML, BioPAX and SBGN are provided by most of the databases. These computer readable files can further be used to develop *in silico* models of pathway through other third party software. In that case, users have to manually include the mathematical rules and kinetic rate parameters, etc. in those file formats.

The data heterogeneity sometimes creates confusion to choose the appropriate and comprehensive data from an exact database. It is therefore developer's responsibility to minimize all the ambiguities and create the database with high level of authenticity and reliability. It can be performed by regular updating of the database with current research outcomes and providing the appropriate cross references to the uploaded reactions data for easy verifications. It is observed that almost all the databases provide the literature references for the uploaded pathway data. Although, except few databases (e.g. BioModels and NETAPTH), the reference(s) for each included interactions of a particular pathway are not much observed in other popular databases and hence the reaction annotations, such as literature references, experimental details, stoichiometry and rate parameters, are not easily available for a specific reaction from these databases. As a result, a large amount of molecular reactions data is still dispersed in many signaling pathway databases without proper annotation. To annotate that data properly, a dedicated database of biomolecular reactions and assignment of a unique identification number of each interaction is required. However, it is already reported that a large number of interaction data available in the PPI databases, such as IntAct (62), Biogrid (36), HPRD (37) and DIP (63), are mostly redundant in nature (64). In this context, iRefIndex, a PPI indexing database, has started to index and assign each reaction ID for all the redundant and non-redundant reactions present in the popular PPIs databases (64), and hence it is believed that using those reaction IDs, the pathway reactions or interactions in the pathway databases can be mapped successfully without any redundancy. Besides, KEGG and REACTOME have also assigned the reaction ID for each reaction of the pathways in their databases. Annotating the pathway interactions data with such database IDs may also help the database users to collate the reaction information with less effort and time. CPDB has also annotated each reaction of a signaling network with the above mentioned PPI databases (Figure 5A).

#### Mutation or disease-related information.

Uncontrolled regulation of several oncoproteins in signaling pathways are found to be involved in various cancers and other diseases (65–68). Hence, a knowledge repository of such oncoproteins associated with various pathways and diseases is essential. There are only two databases, KEGG and Applied Biosystems, which manually annotate the disease-specific information with each protein of the pathways, whereas the databases NetPath, WikiPathways, InnateDB and BIOCARTA simply hyper-link the protein-disease information with another popular disease-specific database OMIM (69). On the other hand, REACTOME has nicely categorized the different diseases with various pathways, which are found to be involved in the progression of those diseases in the experimental results. However, it is observed that except few databases, the annotation of protein-disease information is still not much popular and not uniformly adopted in all of the signaling pathway databases (Figure 5B).

#### Cell or tissue-specific pathway data.

It is experimentally proven that the same signaling pathway can be activated differentially in different tissues or cell lines and thus can exhibit tissue-specific inhomogeneous expression patterns of the target genes or proteins (70, 71). Apart from signaling proteins, there are several factors (e.g. concentration or expressions of miRNA, metabolites, lipids or the intra cellular environment) in cell signaling network, which govern the activity of the pathway and can differentially express in different types of tissue or cell (72–75). Further, it can induce the expression of various proteins and can influence the phenotypic behavior of the tissue. Hence, to get the complete picture of the cell or tissue-specific signaling pathway data, only protein reactions network is not enough, but it is required to include the other tissue-specific factors in that pathway or network. SignaLink provides characterized tissue and cancer-specific protein expressions data freely to the database users. Another popular database, REACTOME, also provides the expressions of the proteins linked with a signaling pathway across different tissues. One can get the expressions of a particular protein in a specific type of a tissue using the ‘expression tab’ linked with each pathway in this database. However, it should be noted that the cell or tissue-specific expression provided by REACTOME is not actively integrated by its curators, rather it is provided through another popular microarray expression database of EBI web resource. Moreover, the gene enrichment analysis present in CPDB can also be used to analyze the genome-wide gene expression or proteome-wide protein abundance analysis for two different phenotypes using Wilcoxon signed-rank test (76). Two other databases (not signaling pathway database), BLOTBASE (77) and Library of Medical Genomics (LoMA) (78), which provide the northern blot results of different tissues, can also be used to map the proteins/genes within a particular signaling pathway (Figure 5C).

#### Data for *in silico* model development.

Several computational models on different cell signaling pathways using various mathematical methods are proposed till now, out of which the mathematical models using the Ordinary Differential Equations, Graph theory, Boolean model, Fuzzy logic, etc. are most popular and widely used (6, 49, 79–83). Interestingly, most of the models are based on the pathway data collated from either the signaling pathway databases or literature mining. Using these data, multi-scale modeling approaches are successfully developed to analyze the signaling pathways more accurately. However, the accuracy of such models rely on several factors such as molecular/protein interactions, concentrations, rate parameters and finally on the accuracy of the constructed reaction network, which are necessary to the model developers. Hence, it is worth to mention that to develop such successful *in* *silico* models, the signaling pathway databases have the scopes to provide the major contributions.

It is found that there are only few databases that store the pathway data relevant for the *in* *silico* model development. BioModels is such kind of database that serves as the repository of the *in* *silico* computational models of the signaling pathways or the biological processes mined from the published literatures. Using Path2Model (84), it automatically generates the mathematical models and enrich it with the cross references. It is a reliable resource for the model developers as it manually curates the model-related data, such as kinetic rate parameters, protein concentration, and dynamic rate equations, from the published literatures. There is another useful database, DOQCS, which serves as the *in* *silico* quantitative model repository for neuron and other signaling pathways. The models provided in this database are collated from peer-reviewed journals and are freely available to the users. The kinetic rate parameters are obtained from various experimental resources, such as enzyme assays, binding experiments and time course of reactions. Both BioModels and DOQCS have authors' assessment, and the users can comment, contact or run the model simulation to test its accuracy in the databases. On the other hand, in REACTOME, user can download the reaction graphs of signal transduction network in various file formats, such as SBML (85), SBGN (20) and BioPAX (19) formats, which provide the computational framework to the users for further *in* *silico* model development. However, it should be mentioned that REACTOME only provides the reactions schema but not the kinetic parameters for quantitative model development (18). Similarly, there are other similar databases like KEGG, NCI-PID, CPDB, NetPath, hiPathDB (86), SignaLink, SPIKE (87), WikiPathways and PANTHER, which also provide the reaction graphs (not quantitative) in various computer readable file formats (e.g. KGML, SBML, SBGN and BioPAX) and by importing these files to specific third party simulation tools such as CellDesigner (88), Cytoscape (89), Bionetgen (90) and Copasi (91), the model developers can incorporate the kinetic parameters, mathematical rules, dynamic equations, initials concentrations, etc. to the reaction graphs and pathway species within the downloaded files and can simulate the pathway models qualitatively or quantitatively (Figure 5D).

It is observed that different types of pathway-related data are present in different signaling pathway databases, and as a result, all such data are still in a scattered situation. However, it is important to note that all these databases have evolved in different time points with new ideas of sharing different types of pathway-related data, new file formats for data sharing purpose, new applications to display the pathway data, and hence, expecting a common and homogenous file formats from the developers of these databases is found to be limited and beyond their scope in this regard. Moreover, the database schema, used by the database administrators to index and store the wide spectrum of pathway information, are also varied in different databases and thus creates the requirement of new file formats to the database developers. Although the requirement of a general platform, including various computational tools and standard file format, to analyze such enormous amount of data from various databases is a general wish to all the database users, however, it is also true that unless all the database developers are agreed upon for a standard file format (like MIAME is being used to share microarray expression data to the genomics community), it is very much difficult to follow certain rules or standard for extensive data curation, storage and sharing process by the database developers. To accelerate the data sharing process and to ease the tremendous efforts, which database developers put on the data curation process, this study claims for the necessity of a general computational and technical platform, through which vast amount of pathway data can be easily accessed from all the available databases and simultaneously can be processed for further pathway data sharing and analysis purposes. To develop such general computational benchmark, it is utmost necessary to understand various technical details, such as the architecture, database schema, file formats and search options, of the existing databases. Hence, this article also evaluates the technical or computational facilities, which are provided in different databases to the general users at free of cost or without any license or agreement.

### Comparison based on technical details

#### Database management system.

To compare the tools and techniques available in the databases, it is first required to compare the database structure and schema as the smoother access of different computational operations is based on these two factors (92, 93). A database schema describes the structure and entire configuration of a database in a specific conceptual language, which is managed by a database management system (93). Different types of database management systems are proposed till now and out of which relational, object or relational object database management systems are widely used and well accepted to most of the database developers (94). Relational Database Management Systems (RDBMS) is implemented in most of the databases by the database drivers made by MySQL, PostgreSQL and Oracle, etc. and various dynamic programming languages, such as PHP and Perl, are used to access and query the data dynamically. On the other hand, there are some databases, which store the data in a simple flat file format, and the data are stored without any object or any rules. Here, the data are simply stored in a text, HTML or XML files and can be searched or parsed by knowing its schema. This type of schema is easy to understand in compared with the RDBMS, but due to the lack of its dynamic property, it is not useful to create dynamic database. Moreover, when heterogeneous elements or objects (i.e. sequence, structure and pictures) are needed to be included into the database, object-oriented schema is more useful in compared with simple flat files. In RDBMS, such type of data can be linked with a common element/pointer and possible to establish one-to-many relationships schema.

While reviewing the database management systems used by the signaling pathway databases, it is observed that most of the databases are based on flat file schema. Initially, the signaling pathway databases were created to host only the pathway diagrams and the description of the pathway. However, as time proceeds, the database becomes more complex as it is started to provide not only the pathway diagram but also the annotation of the pathway objects (protein, gene, RNA, etc.), protein expressions, *in* *silico* or mathematical models, disease-specific information, etc. It is found that databases like CPDB, REACTOME, NCI-PID, Pathway Commons, hiPathDB and BioModels are using object-oriented database schema, and thus, these databases are dynamic in nature. On the other hand, the other databases are mostly focused on the pathway diagrams and provide the data in xml, text and/or image formats. In these databases, the data uploading or updating is the major problem compared with the RDBMS systems. Hence, it is clear that to make an advanced, easily searchable, updateable and accessible database, RDBMS is the best option compared with the other presently use database management system. Using this schema, all the interactions, proteins, their associated information, pathway pictures, etc. can be stored in a tabular format by assigning specific database identification number and can be used to link and parse the data dynamically. Parsing accurate data and to perform complex searches with less time and effort is also a major concern of any database, which can be easily performed by using RDBMS.

#### Search/browse options.

The search options provided in the signaling pathway databases also vary according to the database schema used by the database administrators. There are different types of search options available in different databases; however, it is broadly categorized into two sections: Simple text search and Advance or complex search. Simple text search can be performed by entering simple text or phrase in the search field. It also takes pathway name, pathway ID, biological process, gene or protein name, disease name and sometime multiple terms. This type of simple text-based search option can be included in any types of data structures, although it takes more time if the data is not well structured and indexed in the database. On the other hand, advance or complex search is more computationally rich wherein users can filter or restrict the search terms and run multiple queries at a time to get more specific information from the database. For example, to get the drug names against a specific pathway or protein and have specific side effects, user can run a complex query in the database, through which it will get all the drugs or drug like molecules that target the specific pathway or a protein molecule including their side effects. In Supplementary Table S2, different types of search options used by different databases are provided.

Simultaneously, browsing the pathway names is also an important feature, which is required for searching or selecting a particular pathway from the list of available pathways. It is observed that there are multiple ways adopted by the database developers through which a database can be browsed and out of all browsing by alphabetical or by pathway accession number is most popular, followed by the species/organism-specific browsing. There are other types of browsing options, such as Cellular or Biological Process, Disease names, extracellular signaling molecule/ligand or Category of Pathway-specific browsing, available, through which the databases can also be browsed. In BioModels and WikiPathways, the pathway data are categorized according to their curation level, i.e. the featured pathways and curated pathways, and thus it also provides an extra dimension to browse and select the pathways (see Supplementary Table S2).

#### File formats.

As mentioned earlier, there are multiple types of file formats that are being used by the database developers to provide the pathway data for the database users. Broadly, the types of data present in signaling pathway databases can be categorized in two parts: Pathway Image and Pathway Description. The pathway image is provided in various file formats, such as PDF, PNG, TIFF, JPEG and SVG, and out of all SVG has better advantage compared with the other formats as it is xml and vector-based image file and is easier to zoom in higher magnification level without losing the image quality. Most importantly, it is easier to update by including a new pathway component without redrawing the image diagram. As it is a text-based XML file, therefore to update it, a computer program can be used, which will parse and update the XML tags. Any modern web browser supports this image file, and hence, it is easy to host in the database without providing any image processing software or tool.

On the other hand, the pathway descriptions are mostly provided in SBML, BioPAX, PSI-MI and SBGN (21, 95) file formats. Importance of such standardized file formats is great in respect to the digitization of pathway data, *in* *silico* simulation process and to speed up the user-friendly pathway sharing process. It is a general fact that the complexities of the underlying mechanisms of signal transduction networks make it difficult to express the pathway diagram in a regular process diagram, which is commonly used for reconstructing metabolic pathway diagram. Signal transduction networks includes various types of reaction, transportation and conformational changes, which are difficult to annotate, and hence, a standardized format, which is able to clearly depicts such processes in the signaling network, is very much required in this aspect. Moreover, the recent advancement of systems biology research demands the expertise of various disciplines of science such as biology, chemistry, mathematics and computer science and hence, to share and discuss the pathway data among the scholars of these disciplines, a standard file format without having any ambiguity or technical jargons is always helpful. Hence, to overcome such problem, SBGN, a web-based community, SBML and BioPAX are developed and made available freely to all the users. The purpose of all these formats is to provide the standard graphical notations and language to describe the biochemical pathways unambiguously among a wide range of pathway database users. SBGN and BioPAX are mainly used for pathway visualization by using standard graphical notations as process diagram, whereas SBML file can be used for dynamic model generation as it can store the kinetic parameters, stoichiometry of the chemical reactions, model variable names, etc. in the file format by using some defined xml tags. There are also few simulation tools, such as CellDesigner (88), Copasi (91) and Cytoscape (89) available, which can be used to visualize and simulate such file formats. Besides these file formats, simple CSV, text or tab delimited file formats are also used in many databases. In Supplementary Table S3, a comprehensive list of all the file formats, which are used by the different databases, is provided. However, SBML and BioPAX are the most useful file formats till now, as these are xml based, computer readable, easy to access and most importantly, these file formats use specific legends or graphical notations to annotate the pathway species (proteins, ions, complex, genes, etc.) and various reaction processes. On the other hand, the standard graphical notations provided by SBGN are successfully implemented by REACTOME and PANTHER database to show the complex and large pathway diagram in the database web interface. Using the various notations available in SBGN format, these two databases have not only been able to show a large pathway but also been able to show various complex reaction processes such as complex formation, conformational modification of proteins, phosphorylation and compartmentalization of chemical reactions of a signal transduction network. However, it is observed that the pathway image in PDF format and the pathway data in BioPAX, followed by SBML are the mostly used file formats. There are other specific file formats, such as KGML and GENESIS (96), which are solely developed by the databases KEGG and DOQCS, respectively, available for pathway data sharing purpose.

#### Data download and FTP/API service.

Like other open source biological databases, almost all the signaling pathway databases provide the download option of the pathway image and data to the users at free of cost. There are few databases, which also provide API or FTP access through which bulk and computer modulated download is possible. KEGG provides DBGET and its own FTP/API service to download the pathway data programmatically, although bulk download is available through the subscription of FTP service (97). REACTOME, CPDB and WikiPathways use different types of API service to access and download the pathway data in a local machine (53, 98, 99). For example, REACTOME uses two types of API service: SOAP and RESTful to access and download the REACTOME data remotely. However, to use such facilities, users are required to first understand the data model of REACTOME database through the manual available in their web site by specifying manual to use the API. On the other hand, InnateDB uses PSICQUIC-based WEB-API service to access its data, and JAVA-based WSDL API service is used by BioModels (100) and PANTHER to parse its model data in XML and other file formats.

#### Pathway data upload.

Frequent update of pathway components with the recent published data is not always possible to the database developers, as the pathway information is still dispersed into various sources without proper annotation. Hence, it is very much important to involve various pathway curator communities in the database development, such that continuous flow of new information can flourish the database. To do that, both the users and database developers would have to interact with each other. Initially, most of the databases used to provide only the pathway data and the rendered image in the internet, and thus no interaction was possible at that time with the database users. After the advancement of internet, web browsers and computational tools, it is now easier for the users to curate, annotate and upload a new pathway in the database. Development of several online and offline pathway upload and drawing tools have made it possible. Open source initiative taken by WikiPathway has accelerated this process by providing the crowd sourcing facility in its database website (31). It is now agreeable that collating the large-scale signaling pathway data could not be done without crowd sourcing and open source initiatives. Hence, to attract more curators in the pathway data collection, more number of user friendly, easily accessible and open source applications for pathway uploading and drawing purposes are required.

#### Online analysis tools.

It is almost impossible to provide the customized data to the users by the database developers in the database. Hence, there is a need to develop some online tools and applications, which could process, customize and annotate the raw data according to user's choice and simultaneously perform various *in* *silico* simulations. In this context, few initiatives are already taken by different database administrators (Table 1). To compare different types of online analysis tools available in major databases, this comparison has broadly divided the online tools according their functional modes, which are ‘Pathway Uploading tools’, ‘Pathway drawing tools’ and ‘Pathway analysis tools’. Different databases have found to possess different type of tools in their web interface as listed in Table 1. In this table, it is shown that BIOCARTA, WikiPathways and REACTOME are only database, which provide tools to the users or pathway curators to curate and upload the pathway map or pathway-related information in their databases. On other hand, to facilitate the pathway drawing, KEGG, CPDB, PROTEIN LOUNGE, Pathway Commons and SPIKE provide various type of pathway drawing tools (desktop or online version) in their databases. However, the pathway drawing tool ePath3D, provided by PROTEIN LOUNGE database, is only available after purchasing either its desktop or online versions. Interested users can also use their demo version before purchasing this licensed tool. On the other hand, the open access pathway drawing tools provided by CPDB, Pathway Commons, etc. are also very useful in this regard. Open source, desktop application, Chisio BioPAX Editor (ChiBE), provided by PATHWAYCOMMONS database is also very useful to edit, visualize and modify the pathway models using BioPAX format.

**Table 1.** Detailed description of the signaling pathway database and their computational tools

Tools	Name of the database	Remarks
Pathway Data Uploading Tool	BIOCARTA, WikiPathways, REACTOME	These databases have the tools for uploading pathway data in the specific database formats. Users are required to log in ‘WikiPathways’ to upload or edit any pathway information.
Pathway Drawing Tool	KEGG	KegDraw: It is a Java-based application for drawing compound and glycan structures.
	CPDB	It can create the signaling network or map by uploading the pathway interactions in its own format.
	PROTEIN LOUNGE	3D pathway can be created using its ePATH 3D tool. Although it is not free.
	Pathway Commons	It has Cytoscape Plug in to view, edit and analyze the pathway data. It also has a pathway viewer and editor ChiBE, which is linked to it. Another pathway visualization application PCVIZ is also available in this database. It takes a list of genes, and by finding its neighbors, it generates the pathway diagram.
	SPIKE	It has a tool for pathway drawing and visualization purpose.
Pathway Analysis Tool	BioModels	It has online ODE simulation tool to simulate the pathway models.
	KEGG	KegArray: JAVA-based application for microarray data analysis.
	SignaLink	PathwayLinker: It identifies and visualizes the first neighbor interaction network of the queried proteins, analyzes the signaling pathway memberships of the proteins in this subset and provides links to other online resources. Signalog: It can predict novel signaling pathway components on a genomic scale, based on the signaling pathway membership(s) of its ortholog(s) in eight signaling pathways of three intensively investigated species: *Caenorhabditis elegans*, *Drosophila melanogaster* and human.
	SPIKE, CPDB	These databases have the tools for pathway enrichment analysis using micro array or protein expression data.
	PANTHER	Gene List Analysis: This tool is provided in the database to analyze a set of user-defined gene lists and their expression data with PANTHER database. It maps the gene lists to PANTHER ontology and subsequently grouped into various biological process categories, as well as to the biological pathways. It also overlay the analysis results on PANTHER pathway diagrams to visualize the probable functional relationships between genes/proteins with known pathways.PANTHER scoring: This tool is useful for scoring a user-defined protein sequence against the entire PANTHER library of over 38,000 statistical models, based on HMMs to obtain PANTHER classifications and alignments.Moreover, PANTHER has also built various in-house tools for visualizing, downloading and computing the pathway data. It has also put a significant effort to provide the pathway maps using SBGN standard notation on JAVA-based application platform.
	REACTOME	Pathway Analysis Tools: This tool merges various pathway analysis-related tasks to a single portal, through which one can perform the identifier mapping, overrepresentation and expression analysis. Users can provide Uniprot accession list, Gene name list, NCBI/Entrez list, Small molecule (ChEBI or KEGG) list, microarray and metabolomics data for the mapping and expression study against REACTOME database. REACTOME has also in-built web-based tools to visualize the module-based/functional hierarchy-based pathway components and reactions in web interface.

Besides these pathway drawing and pathway editing tools, a huge effort is also observed to develop various pathway analysis tools by different database developers. Almost all the major databases, such as KEGG, REACTOME, PANTHER and CPDB, have various online or desktop applications software to analyze signal transduction data. A Cytoscape plugin, REACTOME FI, is developed by REACTOME to find pathways and network patterns related to various types of diseases including cancer. Moreover, using a single portal developed by this database can also be used to perform multiple tasks such as identifier mapping, overrepresentation and expression analysis of pathway components. To analyze omics data and clustering analysis using hierarchical clustering algorithm, KEGG has also developed a JAVA-based application, called KegArray to interpret high-throughput data derived from microarray, metabolomics and metagenomics study (101). Similarly, PANTHER and REACTOME have also developed various in-house tools based on JAVA Applet or PHP-based script, to visualize and represent the pathway diagram using SBGN/SBML notations in their web interface. PANTHER also provides the pathway analysis tool for functional analysis of genes, statistical overrepresentation and enrichment test of the user-defined gene list on the basis of statistical analysis of GO ontology data (102). The pathway components or the protein molecules included in the curated pathway modules of PANTHER database are also represented by phylogenetic tree and hidden Markov models (HMMs), which can further be used to map with the user-defined IDs of gene/protein list and can be successively map with the PANTHER pathways. If the gene IDs do not match or available in the PANTHER database, then user can also score the gene list against the HMMs library of PANTHER database and can generate PANTHER generic mapping file. For readers' interest, a detailed description of all the available pathway analysis tools is tabulated and summarized in Table 1.

## Signaling database comparisons based on use cases

For the benefit of the readers, and to highlight the interesting technical features available in different databases, a comparative study based on use cases is also performed in this review. Hedgehog and Notch signaling pathways are considered in this case study to analyze the users' experiences during the curation of these pathway data from different databases. It is observed that the databases with RDBMS data structure (e.g. REACTOME, PANTHER, hiPathDB and CPDB) are easily searchable while searching Hedgehog and Notch pathway information by using simple text or key words search (e.g. pathway name, protein/gene name of the pathway and accession number). The advanced search option is also seemed to be useful in this context. To view the pathway images, SVG or PDF format (provided by REACTOME, WikiPathways, NCI-PID, BioModels, etc.) is more suitable than the other file formats, whereas for pathway data sharing, SBML or BioPAX (provided by REACTOME, PANTHER, NetPath, WikiPathways, NCI-PID, BioModels, etc.) is found to be more useful than the other pathway data sharing file formats, as these formats are easily readable by most of the third party software. In case of downloading the pathway data in local machine, direct static download link provided by most of the databases are useful. However, to perform large-scale pathway analysis, automated pathway annotations or to extract the non-redundant pathway data from various resources, dynamic web-based API service (available in KEGG, REACTOME, PANTHER, WikiPathways, CPDB, etc.) is useful (see Supplementary Tables S2 and S3). In case of online or web-based tools (e.g. pathway upload, drawing and analysis application), databases, such as WikiPathways, BIOCARTA, REACTOME, CPDB, KEGG and PANTHER, provide the best user-friendly tools in their web sites. Hence, through this comparison based on the use cases of technical features, it can be concluded that the databases, such as REACTOME, PANTHER, WikiPathways, NCI-PID, BIOCARTA and BioModels, have created a great impact in this field of study (Table 1).

## Limitations and future directions

### Nomenclature of the pathway

As discussed earlier, there is no as such specific guideline for the nomenclature of the signaling pathways in the databases. It is observed that a same pathway (e.g. Hedgehog and Notch) is named in multiple ways in different databases. Hence, the necessity of a simple but more specific guideline which would be used to name and annotate the signaling pathways in the database is required. Like the other naming conventions (e.g. E.C. number and Chemical compound nomenclature), there should also be a proper way to name the pathways with unique identification number. As most of the signaling pathways are initiated by some specific receptors, therefore receptor-based nomenclature or the ontology-based pathway nomenclature would be a reasonable solution. However, the implication of such nomenclature system is always a debatable matter.

### Pathway reconstruction boundary

**Figure 6.**
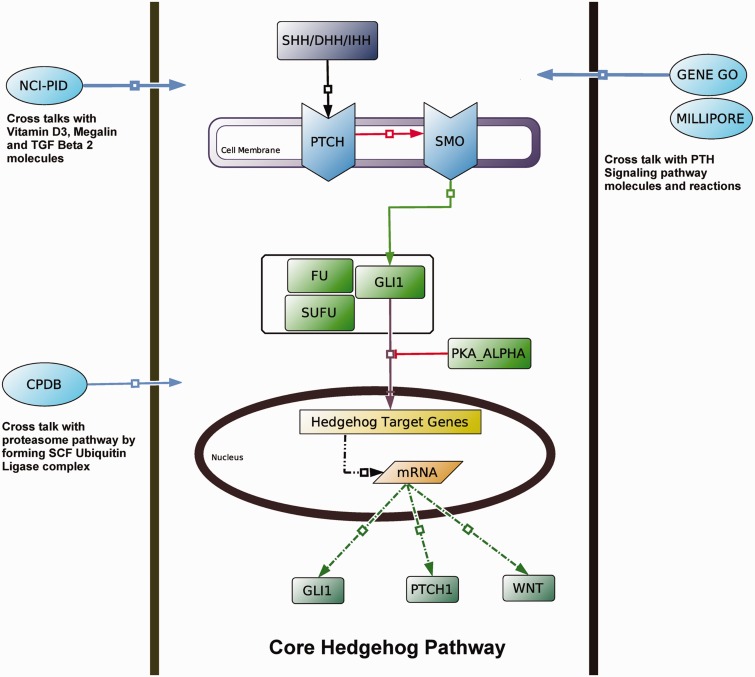
Core Hedgehog pathway and its cross talks. Schematic shows the cross connection of the core Hedgehog pathway (taken from KEGG) with the other pathways and molecules [e.g. Parathyroid hormone (PTH) signaling pathway, proteasome pathway, Vitamin D3, Megalin and TGFβ2 molecules], which are considered in different databases, such as NCI-PID, CPDB, GENE GO and MILLIPORE. Hence, the number of pathway components of this pathway varies significantly within these databases.

It is clearly mentioned in the previous sections that the heterogeneity of the number of molecular species and reactions present in the pathway databases is mostly caused due to the lack of specific boundary conditions for pathway reconstruction. Imposing a boundary to the depth of data curation can restrict the data heterogeneity. It is observed that the inclusion of various cross talking reactions and molecules with the core signaling pathway in different databases brings this heterogeneity. A case study has been performed on Hedgehog signaling network to show how various cross talking molecules and reactions with the core pathway bring such types of data heterogeneity across different databases. For example (schematically represented in Figure 6), GENE GO and MILLIPORE have considered Parathyroid hormone signaling pathway along with Hedgehog pathway to show the mechanism of these two pathways on osteogenic cell proliferation, which influences the bone and cartilage development. NCI-PID shows the cross talk of Hedgehog pathway with the other molecules, such as Vitamin D3, Megalin and TGFβ2. On the other hand, CPDB has shown the proteasome pathway of the Hedgehog pathway molecules. For other signaling pathways, the same situation is also observed where different databases are reconstructing the signaling pathways on their own way. Few have restricted the reconstruction on the core pathway, whereas others are including more information by adding more cross talks with the core pathway molecules.

This heterogeneous pathway reconstruction procedure without any specific boundary or restriction has both merits and demerits. To study the basic mechanisms of a specific pathway, only the core pathway information is sufficient, whereas for experimental or pathway modeling purpose the cross talk information is highly required and useful. In this context, module-based pathway reconstruction can be useful, where the whole pathway will be divided into some functional modules. The core pathway reactions can be allocated into a separate module, and the cross talks would be separated out in the other modules. The modules would be connected by the key molecule, which connects the cross talk reactions with the core pathway. By this way, the database developers would have the freedom to reconstruct and update the pathway by including or updating the modules (i.e. cross talks), and the users will get the module specific or whole pathway information. REACTOME has such kind of hierarchical modules to allocate the pathway in the database. Notch pathway is stored in this database in four parts or modules: Pre-NOTCH Expression and Processing, Signaling by NOTCH1, Signaling by NOTCH2, Signaling by NOTCH3, Signaling by NOTCH4 and Signaling by NOTCH5. Each part is connected with each other, and upon users' preference, it can show the entire pathway or a part of the pathway.

### Inability to show protein complexes

It is a known fact that the signal propagation in signaling pathway through various signaling proteins is not a static process rather it is very dynamic in nature. Various types of protein complexes (transient or stable) are formed during the course of signal transduction, which are found missing in most of the signal transduction information provided by the databases, except REACTOME, InnateDB and NCI-PID. Moreover, the databases are also unable to show the conformational changes of various proteins occurred at the time of pathway activation, except REACTOME, which displays the conformational changes of various proteins using SBGN notations. It is obvious that the static sequential diagrams of signaling pathways provided in the signaling databases are unable to show such complex dynamics of a cell and hence pathway animation would be one of the best solutions in this regard. Besides, by using SBML notations in CellDesigner, one can also incorporate such protein complexes in the pathway diagram, which may cause the diagram look messy, but it would be more information rich when compared with the ordinary wiring or flow diagram of signaling pathways.

### Automatic pathway data curation and annotation

The major problem which most of the signaling pathway databases face till this date is the pathway data curation and its subsequent annotation, which are mostly dependent on active human interaction and intelligence. Right now, there is no tool or computer program available, which can fully automate this process. Several attempts are being taken in the pathway reconstruction procedure, such as various text mining algorithms are used to extract the relevant information from the biomedical journals and patents (103), pathway annotation and illustration software are developed, different ontology-based scoring algorithms are introduced for protein functions (104), etc., but none of the method proves its full proof potentiality in dynamic pathway reconstruction procedure. In this case, better computer programming with logical reasoning, language processing and artificial intelligence are required. Similarly, to annotate the pathway molecules and reactions with the other information or database links, it is very much essential to have an automatic and dynamic tool which will perform the job with more accuracy and less time. To overcome such issues, PathBuilder—an open source web-based application—has received the attention to the pathway curator communities for its advanced feature to annotate biological reaction mechanisms/translocation procedures in the signaling pathways (105), by manually or automatically importing the data from various resources. Using this tool, one can automate the validation of the pathway data formats, import and export the pathway data from various resources according to the required file formats and simultaneously can visualize the corresponding pathway diagrams.

However, the authenticity and accuracy of automatic pathway data curation and annotation is still under the scrutiny of various database developers and end-users. The percentage of accuracy achieved by the fully automated process to reconstruct a pathway diagram is still very low, and hence it is ignored by the most of the database developers. However, to keep the database most up to date with the current research outcomes, it is also required to reduce the entire manual work load and subsequently speed up the whole process by including manual checking process to further verify the automatically curated data. In this context, a semi-automated computer program, which will help to draw, annotate, check, validate and update the pathway resources, could be used by the developers. The software developers should also be encouraged to work on this major issue as there is a huge scope of research still left in this area.

### Automated reconstruction of pathway image

Regular update of the pathway diagram by the newly identified species or reactions is a major concern to the database developers. Usually, most of the database provides the pathway diagram in a raster file formats (PNG, JPEG, etc.) and further modification of such file is a repetitive job. Annotating the pathway molecules in the diagram by its cellular locations, providing the appropriate legend for different molecular entities and biochemical reactions, etc. are also a major concern and require intense human interaction. Therefore, to automate the reconstruction of pathway diagrams, one should use the XML-based dynamic image file, such as SVG (Scalable Vector Graphics), which is easily updateable, easy to host in any modern web browser and easy to convert in any of the raster file format by proper converter tool. A dedicated database using RDBMS can be created where all such type of pathway drawing-related information and relevant updates (i.e. molecule and reactions legends, color code, cellular locations and hyper links) can be stored and depending on user's request the information can be fetched from the database to generate a new SVG file to be served to the user's browsers. Hence, the users will always get up-to-date pathway image, and the developers need not have to redraw the pathway images repeatedly. The quality of such vector image is also good when compared with the raster image, as it can be zoomed or magnified up to many folds without distorting the figure's objects.

### Absence of a computational frame work

Developing, testing and validating hypothesis by *in* *silico* analysis using the pathway data available in the databases is the major focus nowadays in systems biology research. Performing such analysis successfully requires the integration of various working modules (such as pathway curation, standardization, model development and model validation) by the theoretical biologists of this field. Moreover, the operation of such *in* *silico* analysis requires the involvement of various interdisciplinary scientific communities and hence a common computational platform, which will provide and integrate all such working modules, altogether is absolutely required. Therefore, inclusion of such common computational platform in the database web interface is now one of the major challenges to the current database developers. There are few databases, which started to integrate different types of third party computational tools (e.g. CellDesigner, Cytoscape, Copasi and Biolayout) in the database interface. However, the operation of such applications slows the regular functions of the database as most of them are based on JAVA applet and sometimes demands the knowledge of high-tech computational ability from the users. On the other hand, simple PHP or other dynamic language-based computer applications to perform various computational operations with step wise manual can resolve this problem, which is also easy to implement in the database web interface. These types of applications should be made in such a way that it can act as an integrated part of the database, and various computational tasks can be performed simultaneously with less effort and time. For example, Pathway Commons have nicely built a common platform to perform various pathway analysis tasks in the database web interface. More details are provided in the ‘Online Analysis Tools’ section and Table 1. PANTHER and REACTOME are also developing such computational platform to attract large number of end-users to perform the in silico and/or data analysis of wet lab experiments. Few tools have been developed by different databases, (e.g. KegArray by KEGG and online tools by CPDB for microarray data analysis), but a concerted effort to bring all the useful tools for pathway analysis in a common platform is still missing. Moreover, except these few databases, there is no significant development observed in the other databases to develop a common computational platform dedicated for data curation, annotation and large-scale pathway analysis process. In this context, the secondary or aggregator databases and hybrid type databases would have the better opportunities as they collate pathway data from various resources and that data can be successively fed into the online computational tools to perform various tasks.

## Summary and conclusion

Very few review articles are published till now, which mainly emphasize on human cell signaling databases. Moreover, previous review articles often focus only on the six major signaling databases and their comparisons, and studied mainly in the perspective of development of mathematical models or the ease of searching and extracting data at the time of data curation by the database users (14, 17). However, there are several other aspects, which are still remained untouched, such as nature of cell signaling pathway data and availability of other pathway-related information (e.g. disease, tissue or cell-specific protein expressions data for *in* *silico* simulation), annotation or indexing of pathways, pathway data curation process, wide data heterogeneity across different databases, etc. Similarly, from technical point of view, the previous reviews did not elaborately reviewed the technical features such as database schema or management systems that are used by the databases, file formats of the pathway data, searching or browsing options, data download and FTP/API services, etc.. Although there are various studies on cell signaling network available, which report some limitations regarding large-scale data integration from various pathway resources, and simultaneously compare their current constraints (6, 14, 15, 106), but a dedicatedly compiled and a comparison including a wide spectrum of signaling databases and their several features, is still missing. To address this challenge, and by considering the previous reviews in this area, a total 24 active biochemical pathway databases containing the human cell signaling pathway data, are extensively compared and reviewed in this article. This study attempts to resolve all the above mentioned issues and examines comprehensively the evolution and enrichment of the databases along with comparison based on two major characteristics, pathway information and technical details, thereby providing a thorough understanding about the signaling pathways information available in the existing databases. This review not only helps to identify some novel and useful features, which are not yet included in any of the databases but also analyze the current situations of the databases with respect to the present requirements in biological research.

Further, some reasonable solutions to the challenges or constrains are identified and proposed in this review. These proposed solutions may help the future database developers to design their database with enriched information and user-friendly options. Experimental and theoretical biologists will also gain some insights about these widely used databases and can plan to choose the proper database for further analysis on cell signaling network. Moreover, this review article gives a brief overview of different types of data (e.g. disease, cell-specific protein expressions data and different types of signaling pathways), which are available in different databases. It will help the users to select the appropriate database and to curate the data easily and accurately.

## Supplementary data

Supplementary data are available at *Database* Online.

Supplementary Data
