# Supplementary material for: Comparison of human cell signaling pathway databases—evolution, drawbacks and challenges
Source: Database (Oxford). 2015 Jan 28;2015:bau126. doi: 10.1093/database/bau126 (PMC4309023; doi:10.1093/database/bau126)
Supplement: Supplementary Data [file supp_2015_bau126_index.html]

Comparison of human cell signaling pathway databases—evolution, drawbacks and challenges — Supplementary Data 

# Comparison of human cell signaling pathway databases—evolution, drawbacks and challenges

## Supplementary Data

files

**Files in this Data Supplement:**

- Supplementary Data - docx file
