# Supplementary material for: Comparison of human cell signaling pathway databases—evolution, drawbacks and challenges
Source: Database (Oxford). 2015 Jan 28;2015:bau126. doi: 10.1093/database/bau126 (PMC4309023; doi:10.1093/database/bau126)
Supplement: Supplementary Data [file supp_bau126_SupplementaryData_DATABASE-2014-0123R1.docx]

**Supplementary Data**

**Table S1. Name and HTTP links of the Databases considered in this review.**

| **Name** | **HTTP Link** |
| --- | --- |
| **Commercial Human Cell Signaling Database** | |
| GENEGO: PATHWAY MAPS | http://pathwaymaps.com/maps/ |
| BIOCARTA | http://www.biocarta.com/ |
| PROTEIN LOUNGE | http://www.proteinlounge.com/ |
| Cell Signaling TECHNOLOGY | http://www.cellsignal.com/index.jsp |
| MILLIPORE | http://www.millipore.com/pathways/pw/pathways |
| Applied Biosystems | http://www5.appliedbiosystems.com/tools/pathway/ |
| INVITROGEN | http://www.invitrogen.com/site/us/en/home/Products-and-Services/Applications/Cell-Analysis/Signaling-Pathways.html |
| **ACADEMIC HUMAN CELL SIGNALING DATABASE** | |
| KYOTO ENCYCLOPEDIA OF GENES AND GENOMES (KEGG) | http://www.genome.jp/kegg/ |
| SIGNALING PATHWAY DATABASE (SPAD) | http://www.grt.kyushu-u.ac.jp/spad/ |
| DOQCS | http://doqcs.ncbs.res.in/ |
| REACTOME | http://www.reactome.org/ReactomeGWT/entrypoint.html |
| PATHWAY INTERACTION DATABASE (PID) | http://pid.nci.nih.gov/ |
| CPDB | http://cpdb.molgen.mpg.de/ |
| NetPath | http://www.netpath.org/ |
| Pathway Commons | http://www.pathwaycommons.org/about/ |
| hiPathDB | http://hipathdb.kobic.re.kr/browse.php?dbType=1 |
| SignaLink | http://signalink.org/ |
| SPIKE | http://www.cs.tau.ac.il/~spike/ |
| WikiPathways | http://wikipathways.org/index.php/WikiPathways |
| InnateDB | http://www.innatedb.com/ |
| INOH | http://inoh.hgc.jp/inohblog/main/ |
| BioModels | http://www.ebi.ac.uk/biomodels-main/ |
| GOLD.db | https://gold.tugraz.at/ |
| PANTHER | http://www.pantherdb.org/ |

**Table S2. Name of the databases and their corresponding search and browsing options.**

| **Search Option** | **Database Names** | **Required fields/ Browsing options** |
| --- | --- | --- |
| **Simple Text Search** | Pathway Commons, BIOCARTA, PROTEIN LOUNGE, Cell Signaling TECHNOLOGY, INVITROGEN, hiPathDB, SignaLink, PANTHER | Simple text or phrase |
|  | Applied Biosystems | Gene ID, Gene Name, Gene Symbols, NCBI Transcript, Pathway keywords, Pathway name |
|  | NCI-PID | Molecule name/ID or biological process term/ID |
|  | NetPath | Gene Symbol, Protein Name, Database Accession Number, Pathway Name |
|  | DOQCS | Accessions, Pathway, Molecule, Enzyme, Reaction names |
| **Advanced Search** | MILLIPORE | Multiple Text terms, Cellular process and Disease area |
|  | InnateDB | Organisms, Protein/Gene name, InnateDB Molecule ID, HUGO Gene Symbol, HUGO ID, Gene Ontology (GO) Accession, Gene Ontology (GO) Term, Ensembl ID, UniProt Accession, Entrez Gene ID, RefSeq Accession, UniGene ID, OMIM ID, EMBL Accession, PFAM Accession, InterPro Accession |
|  | BioModels | BioModels ID, authors names, SBML elements, Gene Ontology, Taxonomy, ChEBI, Publication, Pubchem compounds, Other database ID etc. |
|  | CPDB | Pathway name, Pathway accession number, Physical entity name, Physical entity accession number |
| **Browse Option** | KEGG, WikiPathways, PROTEIN LOUNGE, Applied Biosystems, INVITROGEN, NCI-PID, NetPath, hiPathDB, SPIKE, InnateDB, GOLD.db, Cell Signaling TECHNOLOGY | By Pathway Names/ Alphabetical Order |
|  | WikiPathways, REACTOME, DOQCS, INOH, GOLD.db | By Species Name |
|  | MILLIPPORE, REACTOME, GENEGO, BIOCARTA | By Cellular or Biological Process |
|  | MILLIPPORE, NetPath | By Disease names |
|  | WikiPathways, PROTEIN LOUNGE | By collection (eg. Featured Pathway, Curated collection, etc.) |
|  | SPAD, PID, SPIKE, Cell Signaling TECHNOLOGY | By extracellular signalling molecule/ligand or Category of Pathway |
|  | DOQCS, hiPathDB | By Database ID, Cellular compartment, Tissue |

**Table S3. Types of file formats used in different signaling pathway databases**

| **File Formats** | **Database Name** |
| --- | --- |
| **Image File** | |
| PDF | Cell Signaling TECHNOLOGY, REACTOME, WikiPathways, BioModels |
| PNG | GENE GO, REACTOME, WikiPathways |
| JPEG/JPG | NCI-PID |
| SVG | NCI-PID, WikiPathways, BioModels, GOLD.db |
| GIF | BIOCARTA, SPAD |
| **Pathway Data** | |
| SBML/SBGN | REACTOME, NetPath, SignaLink, BioModels, PANTHER |
| BioPAX | REACTOME, NCI-PID, NetPath, Pathway Commons, SignaLink, SPIKE, WikiPathways, BioModels, PANTHER |
| PSI-MI | CPDB, NetPath, SignaLink |
| CSV | SignaLink, InnateDB, BioModels |
| MATLAB | BioModels |
| OCTAVE | BioModels |
| EXCELL/XLS | Applied Biosystems, NetPath, InnateDB |
| TAB DELIMITED | CPDB, NetPath, REACTOME, WikiPathways, InnateDB |
| KGML | KEGG |
| GENESIS | DOQCS |
| XML | NCI-PID, InnateDB |
| MySQL database dump | hiPathDB, REACTOME |
| SIF | SPIKE, InnateDB |
